# Supplementary material for: Absence of relevant QT interval prolongation in not critically ill COVID-19 patients
Source: Sci Rep. 2020 Dec 8;10:21417. doi: 10.1038/s41598-020-78360-9 (PMC7722753; doi:10.1038/s41598-020-78360-9)
Supplement: Supplementary file 1 — Supplementary Information 1. [file 41598_2020_78360_MOESM1_ESM.pdf]

SUPPLEMENTARY MATERIAL

TITLE

Absence of relevant QT interval prolongation in not critically ill COVID-19 patients.

Juan Jiménez-Jáimez,[1,2]*, Rosa Macías-Ruiz[1,2], Francisco Bermúdez-Jiménez[1,2,3], Ricardo Rubini-Costa[1,2], Jessica Ramírez Taboada[1,2,4], Paula Isabel García Flores[1,2,5], Laura Gallo Padilla[1,2,4], Juan Diego Mediavilla García[1,2,4], Concepción Morales García[1,2,5], Sara Moreno Suárez[1,2,6], Celia Fignani Molina[1,2,6], Miguel Álvarez López[1,2] and Luis Tercedor[1,2]

[1]Cardiology Department, Virgen de las Nieves University Hospital, Avenida de las fuerzas armadas 2, 18014 Granada, Spain
[2]Biosanitary Research Institute (IBS), Av. Del conocimiento, 18012, Granada, Spain,
[3]Centro Nacional de Investigaciones Cardiovasculares Carlos III (CNIC), 28029, Madrid, Spain,
[4]Department of Internal Medicine, Virgen de las Nieves University Hospital, Avenida de las fuerzas armadas 2, 18014 Granada, Spain
[5]Department of Pneumology, Virgen de las Nieves University Hospital, Avenida de las fuerzas armadas 2, 18014 Granada, Spain
[6]Emergency Department, Virgen de las Nieves University Hospital, Avenida de las fuerzas armadas 2, 18014 Granada, Spain
* jimenez.jaimez@gmail.com

Summarized Protocol of general management and pharmacologic treatment of SARS-CoV-2 in the University Hospital Virgen de las Nieves of Granada, Spain

1. Patient without pneumonia, signs of severe illness, comorbidity and age <50: outpatient management with symptomatic treatment.
2. Patient with comorbidity or age >50 or pneumonia without hospital admission criteria (SpO2 >92%, FINE I/II or CURB65 ≤1), outpatient management with:
    a. Hydroxychloroquine 400 mg BID (loading dose) and 200 mg BID during 5 days
    b. Azithromycin 500 mg SID (loading dose) and 250mg SID during 5 days
    c. Acetylcysteine: 600 mg BID during 5 days.

3. Patient with pneumonia and/or hospital admission criteria:
    a. Hydroxychloroquine 400 mg BID (loading dose) and 200mg BID during 5 or more days at physician criteria based on severity illness.
    b. Azithromycin 500 mg SID (loading dose) and 250mg SID during 5 days, or 500 mg iv SID the day 1, 2 and 4.
    c. Acetylcysteine: 600 mg oral/iv BID during 5 days.
    d. Lopinavir/Ritonavir 400/100 mg BID or Darunavir/Ritonavir 600/100 mg BID, during 7 to 14 days at physician criteria based on severity illness.
4. Torpid evolution. severe illness and without criteria for ICU admission:
    a. Hydroxychloroquine 400 mg BID (loading dose) and 200mg BID during 5 or more days at physician criteria based on severity illness.
    b. Azithromycin 500 mg SID (loading dose) and 250mg SID during 5 days. or 500 mg iv SID the day 1, 2 and 4.
    c. Acetylcysteine: 600 mg oral/iv BID during 5 days.
    d. Lopinavir/Ritonavir 400/100 mg BID or Darunavir/Ritonavir 600/100 mg BID, during 7 to 14 days at physician criteria based on severity illness.
    e. If analytic signs of proinflammatory response:
        i. Moderate proinflammatory response: methylprednisolone 0.5 mg/kg iv BID during 3-5 days.
        ii. Severe proinflammatory response: bolus of methylprednisolone 500 mg iv (first day) and 0.5 mg/kg iv BID during 3-5 days and Tocilizumab should be considered.

5. Torpid evolution during 3-5 days despite of treatment with previous step: alternative treatments should be considered such as Sirolimus, Ciclosporin A, immunoglobulin therapy and other monoclonal antibodies and anti-cytokines treatment.

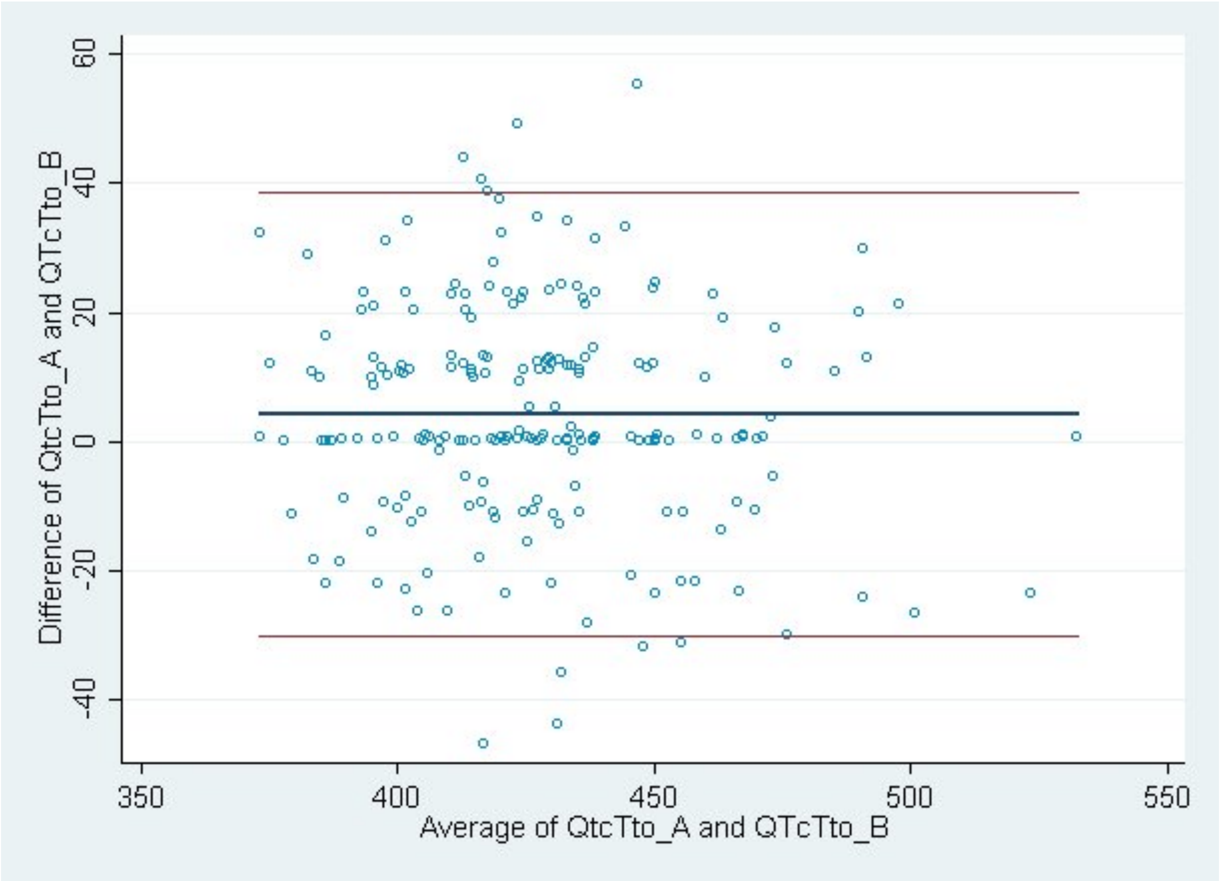

Supplementary Figure 1. Blant-Altman test showing a high grade of concordance between both observers

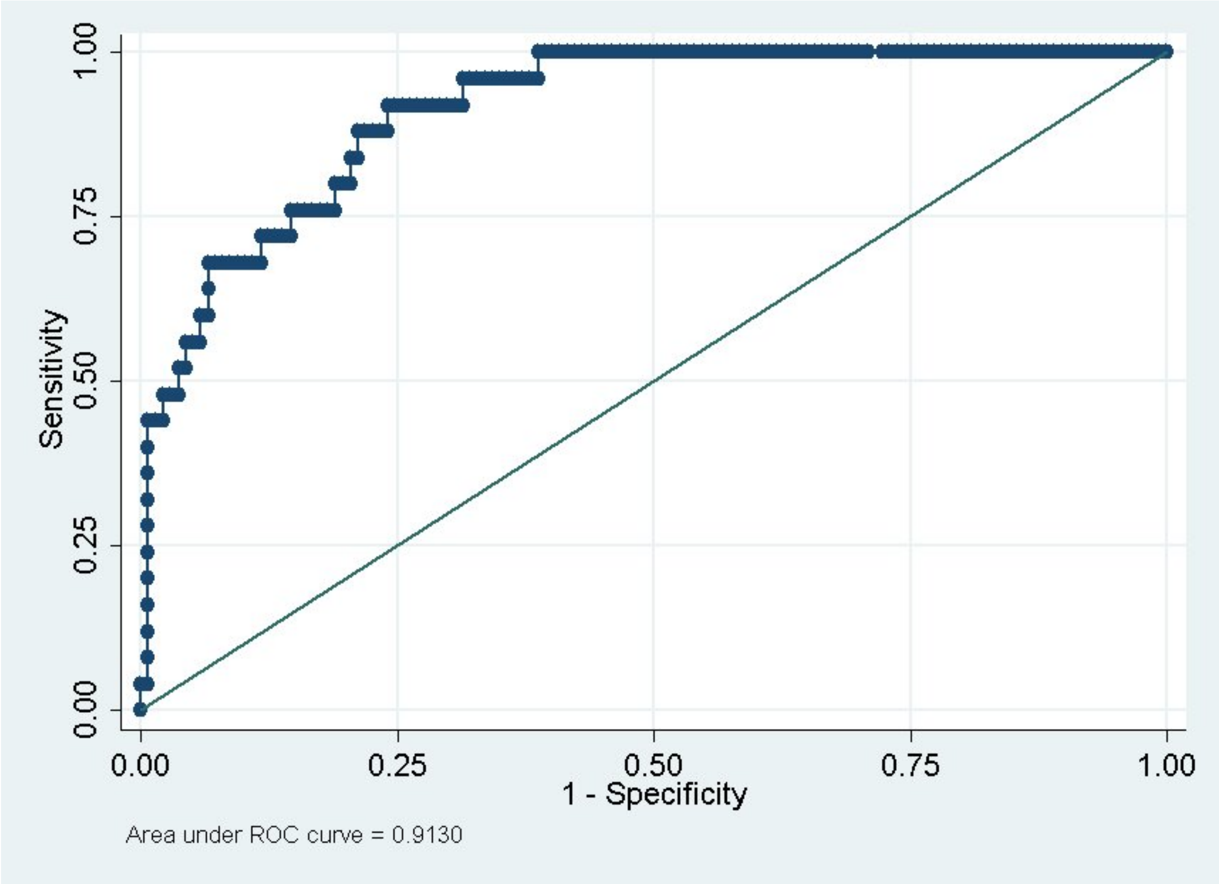

Supplementary Figure 2. Predictive ability of the multivariate analysis, area under the ROC curve.
